# Development of an effective method utilizing fibrin glue to repair pleural defects in an ex-vivo pig model

**DOI:** 10.1186/s13019-020-01158-3

**Published:** 2020-05-24

**Authors:** Nobuyuki Kondo, Yoshitaka Takegawa, Masaki Hashimoto, Seiji Matsumoto, Shiro Oka, Seiki Hasegawa

**Affiliations:** 1 Department of Thoracic Surgery, Hyogo College of Medicine, 1-1 Mukogawa, Nishinomiya, Hyogo 663-8501 Japan; 2 KM Biologics Co., Ltd., 1341-1 Kyokushi Kawabe, Kikuchi-shi, Kumamoto, 869-1298 Japan

**Keywords:** Pleural defect, Air leakage, Fibrin glue, Lung-sparing surgery, Under ventilation

## Abstract

**Background:**

The present study aimed to use an ex-vivo model to investigate whether a new method involving the use of fibrin glue and a polyglycolic acid (PGA) sheet under ventilation enhances the sealing effect after repair of the pleural defect.

**Methods:**

Ex-vivo pig lungs were used in this study. We investigated the maximum pressure tolerance of pleural defects repaired using three methods: 1, directly spraying fibrin glue over a PGA sheet; 2, spreading fibrinogen on the site then sealing with a PGA sheet and spraying with fibrin glue; and 3, spreading fibrinogen while maintaining ventilation then sealing with a PGA sheet and spraying with fibrin glue.

**Results:**

The maximum tolerable pressures were as follows (mean ± standard deviation, cmH_2_O): Method 1, 37.1 ± 13.6, Method 2, 71.4 ± 27.7, Method 3, 111.5 ± 8.8. Histological findings explained the difference in tolerable pressure at the repaired site between methods. Microscopic findings of lungs repaired using Method 3 indicated that the fibrinogen penetrated into deeper tissues to act as an anchor.

**Conclusions:**

Fibrin glue sealing under ventilation increases the anchoring effect of repairing air leakages due to pleural defect in an ex-vivo model. This method may have clinical application. For example, it may be useful to reduce severe air leakage in patients who undergo lung-sparing surgery for a pleural tumor.

## Background

Air leakage remains a major complication of pulmonary surgery which can lead to prolonged drainage and severe complications such as empyema [1]. Fibrin sealant, in combination with bioabsorbable mesh, can be used to create a good seal following pulmonary surgery, and this combination is routinely used in clinics [2–4]. Recently, a new adhesive with the potential to replace blood products such as fibrin glue has shown promise in animal experiments [5].

In our clinical experience of pleurectomy/decortication (P/D) for pleural mesothelioma, we discovered that this combined technique is insufficient to control air leakage. All of the visceral pleura is removed during P/D surgery, meaning that conventional methods cannot control air leakage sufficiently, and long-term postoperative thoracic drainage, pleurodesis, and re-operation may be required. More efficient methods are needed in order to repair air leakage after pleural resection such as P/D; for which, the use of fibrin glue and polyglycolic acid (PGA) sheets shows potential [6, 7].

Several different techniques involving fibrin glue and PGA sheets have been reported [3, 8–10]. Some studies have demonstrated the utility of an optimized method of rubbing lung tissue with a fibrinogen solution and then placing a PGA sheet over the rubbed area [8, 11]. Histology has revealed the tissue penetration of fibrin glue to act like an anchor between the PGA sheet and lung parenchyma, which may explain the excellent adhesive strength of this combination [3, 9, 10].

We routinely perform lung ventilation to test the sealing after repair of an air leakage during pulmonary surgery. Therefore, in the present study, we examined both the conventional method and a novel method of sealing air leakage. The novel method aims to enhance the penetration of the fibrin glue by ventilating the lungs while rubbing the fibrinogen solution onto the lungs. This new method was tested using an ex-vivo model, and may provide an alternative approach to repairing pulmonary leakage, especially after P/D.

## Methods

### Ex-vivo model

Ex-vivo pig lungs were used for this study. Commercially available pig lungs (Tokyo-Shibaura Zouki Co. Ltd., Tokyo, Japan) were obtained, and we prepared a protocol to create the model in a laboratory. The ex-vivo model was created by a single surgeon, to replicate resection of the visceral pleura in lung-sparing surgery for malignant pleural mesothelioma. We used lungs obtained from dead animals; therefore, ethics approval and application procedures to the Animal Experimentation Review Board were not required.

### Creation of the pleural defect

**Fig. 1:**
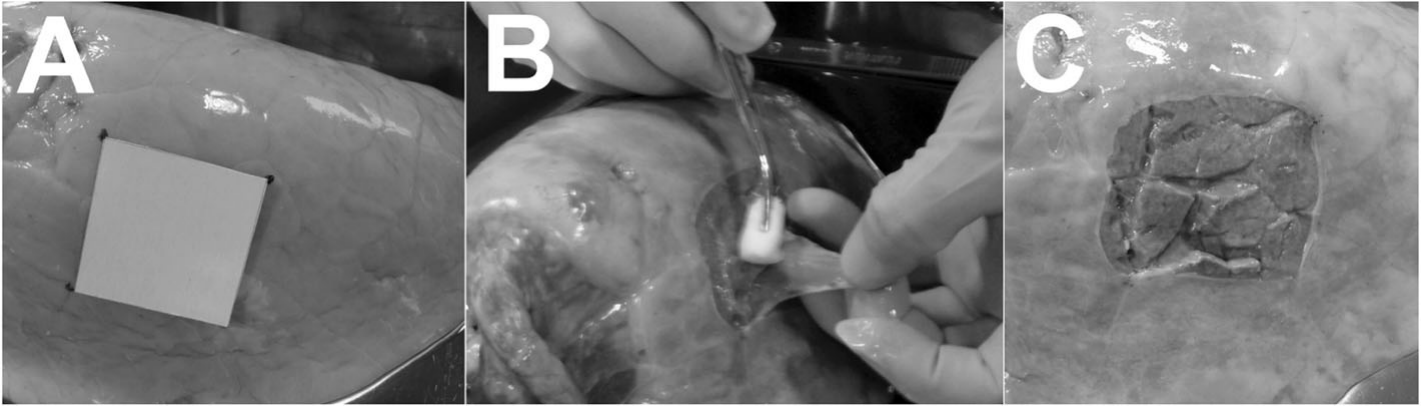
The development of an ex-vivo pig lung model with a pleural defect. An ex-vivo pig lung model was created as follows: **a** A 5-cm square mark was made on the lung surface. **b** The visceral pleura was peeled off with Tupfer gauze. **c** A pleural defect was created in the 5-cm square by incising the visceral pleura with a scalpel

A balloon was inserted into the bronchus of an isolated porcine lung and connected to a nitrogen gas cylinder and pressure gage. A pleural defect (5 × 5 cm) was created by marking a 5-cm square on the lung surface (Fig. 1a) and incising the visceral pleura with a scalpel. While holding one end with forceps or fingers, the visceral pleura was peeled off with a Tupfer gauze to create a pleural defect within a square (Fig. 1b, c).

### Tissue sealants

The materials as tissue sealants used in this study were commercially available. The sealant was fibrin glue (Bolheal®, Chemo-Sero Therapeutic Research Institute, Kumamoto, Japan) comprising fibrinogen (80 mg/ml) blood coagulation factor XIII (75 units/ml), bovine aprotinin (1000 units/ml), thrombin solution (250 units/ml), and calcium chloride (5.9 mg/ml). The tissue-absorbable sheets were composed of 0.15 mm thick PGA polymer (Neoveil®, Gunze Ltd. Co., Kyoto, Japan).

### Sealing method

The pleural defect was repaired using fibrin glue and a PGA sheet (7 × 7 cm). Three methods of repairing lung defects were trialed. Method 1: mixed fibrin glue (2.5 ml) was sprayed over the PGA sheet covering the site of the pleural defect. Method 2: 1 ml of fibrinogen solution was spread onto the site using the finger-rubbing method [11] and sealed with the PGA sheet; then, mixed fibrin glue (1.5 ml) was sprayed over the sheet. Method 3: 1 ml of fibrinogen solution was spread as in Method 2, with gentle ventilation applied for five breaths during the procedure at a maximum airway pressure of 15 cmH_2_0. The defect was then sealed with a PGA sheet and mixed fibrin glue (1.5 ml) sprayed over the sheet. The third method was the procedure that was developed in this study, with the aim of improving the repair of air leakage after the removal of visceral pleura. We named this method the ventilation-anchoring method (VA method).

### Measurement of the seal-breaking pressure

Three minutes after the pleural defect was repaired, the highest pressure in the airway governing the repair site was measured. Nitrogen was slowly fed into the bronchus from the tube connected to the balloon, and the airway pressure when the sealing of the repair site failed was recorded as the maximum pressure resistance. The polymerization reaction of the fibrin glue product occurs within 3 min (in-house data, Kaketsuken); therefore, pressurization was initiated 3 min after repair, and a pressure test carried out every 3 min thereafter applying the mixed spray which included thrombin. This was carried out for lungs repaired by each method (*n* = 4 per method, One sample for each sealing-procedure).

### Pathological evaluation

An experimental model with the pleural defect was created separately from the pressure test and was repaired using each method. To perform histopathological analysis, tissues including the lung parenchyma at the repaired site repaired by each method were excised and fixed in a 10% formaldehyde solution. The fixed specimens were embedded in paraffin, sectioned, and stained with hematoxylin and eosin for microscopic examination. Six specimens were prepared for each method. Representative histological findings are presented.

### Statistical analysis

Data of seal-breaking pressure for each method were analyzed using the Wilcoxon rank sum test. Statistical analysis was performed using JMP Pro version 14.2 (SAS Institute Inc., Cary, NC, USA). All data are presented as the mean ± standard deviation. Statistical significance was accepted at *p* < 0.05.

## Results

### Seal-breaking pressure in the ex-vivo model

**Fig. 2:**
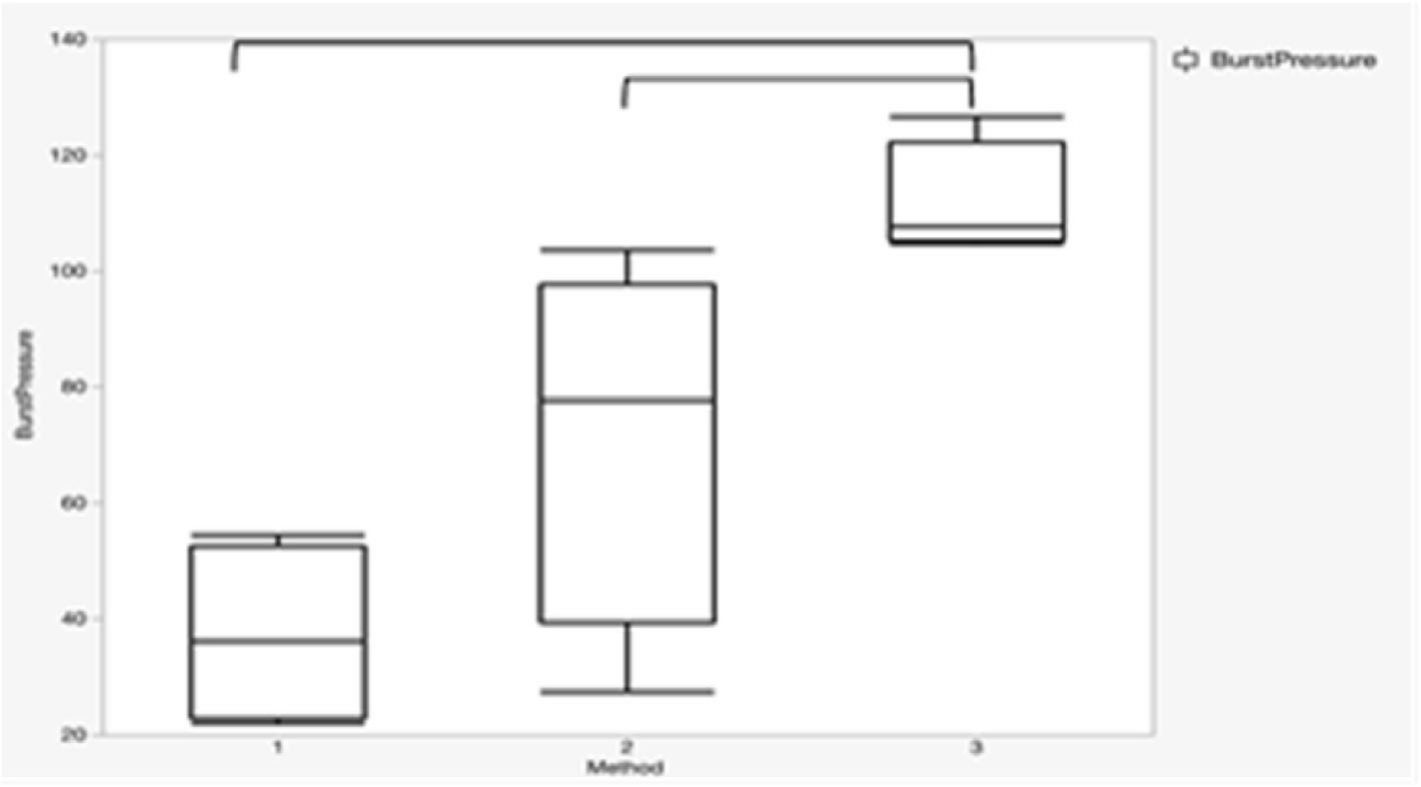
Box-whisker plots illustrating the seal-breaking pressure of ex-vivo lungs repaired by each method. There was a significant difference between lungs repaired using Methods 3 and 2 (*p* = 0.03) and Methods 3 and 1 (*p* = 0.03). Data were analyzed using the Wilcoxon rank sum test. In three out of the four cases repaired using Method 3, pressure measurements ceased because the intact pleura burst before the repaired site failed. Method 1: sprayed mixed fibrin glue over the PGA sheet. Method 2: using the finger-rubbing method, sprayed fibrinogen solution over the seat. Method 3: with gentle ventilation, the finger-rubbing method was performed and sprayed fibrin glue over the seat

The maximum pressure resistance values of the lungs repaired using Methods 1, 2, and 3 are summarized in Table 1. Among these, the mean maximum pressure value was the highest for the lungs repaired by Method 3 (Fig. 2). There was a significant difference between Methods 2 and 3 (*p* = 0.03); however, there was no significant difference between Methods 1 and 2. In three out of the four lungs repaired using Method 3, pressure measurements were stopped due to bursting of the intact pleura before the repaired site failed.

**Table 1:** Seal-breaking pressure values of the ex-vivo models

	Method 1	Method 2	Method 3
1	54.4	80.2	106.1
2	25.8	27.2	126.5
3	21.8	74.8	108.8
4	46.2	103.4	104.7
mean+/−SD	37.1+/−13.6	71.4+/−27.7	111.5+/−8.8
			(cmH_2_O)

### Histological evaluation

**Fig. 3:**
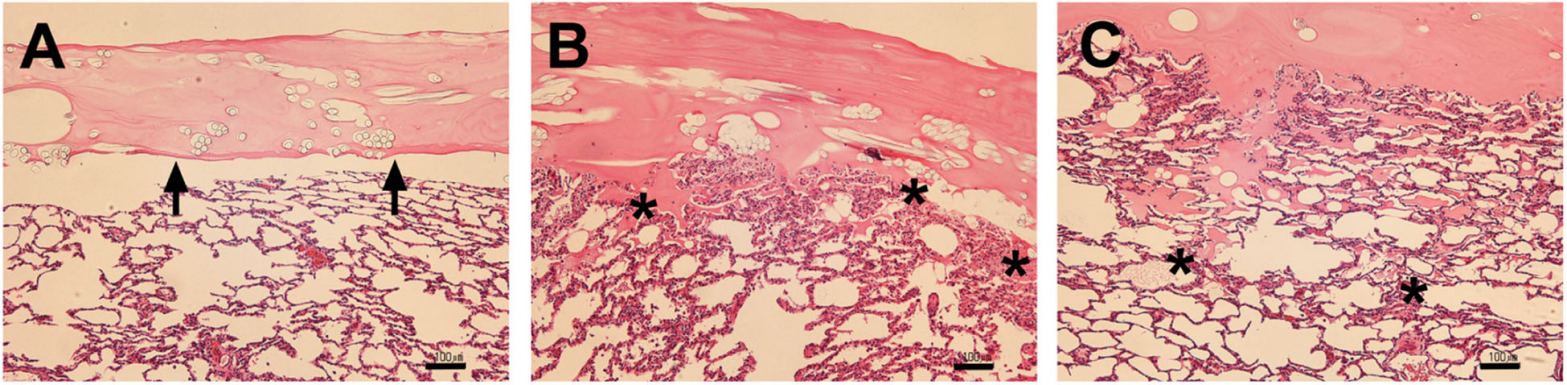
Histological evaluation of tissue from lungs repaired using each method. Specimens of the ex-vivo lung models repaired by each method were stained with hematoxylin and eosin for microscopic observation. **a** Tissue from lungs repaired using Method 1: The fibrin glue does not penetrate the lung parenchyma (indicated by black arrows). **b** Tissue from lungs repaired using Method 2: Partial penetration of fibrin glue into the lung tissue was observed (asterisks). **c** Tissue from lungs repaired using Method 3: Penetration of fibrin glue at the deepest area of the lung parenchyma was observed

The degree of fibrin glue in the alveolar tissue reflected the difference in tolerable pressure at the repaired site for each Method (Fig. 3). For Method 1, fibrin glue was not observed to penetrate the lung parenchyma, nor did it adhere to the uneven surface of the plural defect (Fig. 3a). For Method 2, the PGA sheet was observed to be in close contact with the lung tissue. Partial penetration of fibrin glue into the lung tissue was observed (Fig. 3b). For Method 3, as for Method 2, the PGA sheet was in close contact with the lung tissue. When fibrin glue was identified at the microscopic level, it was found to consistently penetrate to a deeper area of the lung tissue than was observed for the other two methods (Fig. 3c).

## Discussion

The use of fibrin glue and PGA sheets is a routine approach to control air leakage from visceral pleura in thoracic surgery [12]. Methods for repairing air leaks with sufficient strength over a wide area are highly important to ensure the success of P/D surgery [7].

The adhesion mechanism of fibrin involves the action of thrombin, which forms a complex with fibrin to achieve adhesion [13]. Compared with fibrin glue spray, the combination of fibrin glue and tissue-absorbable sheets has proven to be an efficient method for comparing burst pressure in various reports [8, 10, 11]. However, the present study revealed that the withstanding pressure can vary considerably, particularly in Method 2, and is potentially insufficient for clinical use.

The method developed in the present study which involved the use of fibrinogen with constant ventilation was found to increase the pressure tolerance of the repaired lung. We observed a stable pressure resistance, even at pressures that were high enough to cause normal lung tissue to rupture. Histological observations indicated that the new method created deep penetration of fibrin glue into the lung tissue in the area of the pleural defect, with the formation of fibrin clots. These results demonstrate that the application of fibrin solution under ventilation induces the glue to penetrate deeper into the tissue than conventional methods, creating a stronger anchor for the coated PGA sheet. We believe that this is the mechanism underlying the high-pressure resistance that we observed. Histological examination revealed no penetration of fibrin complex into the blood vessels; thus, we consider this method to be as safe as other methods. Due to the histological findings, we named this the VA method to reflect the additional procedure of ventilation and histological observations.

Our results indicate that the use of fibrin glue to seal lung tissue under ventilation can achieve good repair of air leakages after removal of the visceral pleura in an ex-vivo porcine model; the burst pressure of the lung can only be measured with such a model. In lungs repaired using the new method, we measured pressure tolerances that exceeded 100 cmH_2_O. Thus, the repair of pleural defects using the VA method can achieve repairs able to tolerate unexpected intrabronchial pressure, such as that experienced during severe cough after thoracotomy.

Compared with the conventional method (in which solution is rubbed onto the lung tissue [11]), our new method achieved increased pressure resistance according to the burst test using an ex-vivo pleural defect pig model. The effectiveness of pleural repair with fibrin glue and tissue-absorbable sheets was greatly enhanced when performed under ventilation. This finding may enable more effective seals to be created for the repair of air leakages after pleural resection. We have already applied this method in lung-sparing surgery for malignant pleural mesothelioma in our hospital, and have found the outcomes to be preferable to those of conventional methods. Thus, our method could have wide application in thoracic surgery.

This study has some limitations which should be acknowledged. First, the sample size was small, and so further validation is required. With the conventional method (Method 2), the results of the seal-breaking pressure measurement were varied; more accurate statistical analysis could be performed if the sample number were increased. However, for the newly developed method (Method 3), the seal-breaking pressure was relatively consistent. The creation of the model required a stable surgical procedure, so as not to affect the pressure tolerance values. Considering these limitations, we conclude that the findings presented here obtained using a small sample size demonstrated the stability and superiority of the new method.

## Conclusions

Our results demonstrate that pleural defects can be repaired to achieve very high-pressure resistance with fibrin glue and PGA sheets using the VA method which involves rubbing fibrinogen on the defect under ventilation. In the future, we plan to evaluate the application of this method to clinical cases for the surgery of visceral pleural injury or resection.

## Data Availability

All data generated and analyzed during this study are included in this published article.

## Abbreviations

PGA: Polyglycolic acid
VA: Ventilation-anchoring
